# Effectiveness of Long-Term Opioid Therapy for Chronic Pain in an Outpatient Palliative Medicine Clinic

**DOI:** 10.1089/jpm.2023.0251

**Published:** 2024-01-05

**Authors:** Chad D. Kollas, Kevin Ruiz, Amy Laughlin

**Affiliations:** ^1^ Supportive and Palliative Care, Orlando Health Cancer Institute, Orlando, Florida, USA.; ^2^ Breast Medical Oncology and Cancer Genetics, Orlando Health Cancer Institute, Orlando, Florida, USA.; ^3^ University of Central Florida College of Medicine, Orlando, Florida, USA.

**Keywords:** cancer pain, chronic pain, opioid therapy, outpatient palliative care, pain policy

## Abstract

**Background:**

Despite widespread use of opioid therapy in outpatient palliative medicine, there is limited evidence supporting its efficacy and safety in the long term.

**Objectives:**

We sought to improve overdose risk scores, maintain pain reduction, and preserve patient function in a cohort with severe chronic pain as we managed opioid therapy for a duration of four years in an outpatient palliative care clinic.

**Design:**

Over four years, we provided ongoing goal-concordant outpatient palliative care, including opioid therapy, using quarterly clinical encounters for a patient cohort with chronic pain.

**Setting/Subjects:**

The project took place in the outpatient palliative medicine clinic of a regional cancer center in Orlando, Florida (United States). The subjects were a cohort group who received palliative care during the time period between July 2018 and October 2022.

**Measurements:**

Key metrics included treatment-related reduction in pain intensity, performance scores, and overall overdose risk scores. Secondary metrics included cohort demographics, average daily opioid use in morphine milligram equivalents and categorization of type of pain.

**Results:**

In 97 patients, we observed a stable mean treatment-related reduction in pain intensity of 4.9 out of 10 points over four years. The cohort showed a 2-point (out of 100) improvement in performance scores and an 81-point (out of 999) reduction in mean overall overdose risk score.

**Conclusions:**

We present evidence that providing outpatient palliative care longitudinally over four years offered lasting treatment-related reductions in pain intensity, preservation of performance status, and reduction in overall overdose risk.

## Background

Calls to improve end-of-life care in the 1990s expanded hospice and palliative medicine (HPM),^1^ but also contributed to increased opioid prescribing that would be described as an “opioid epidemic” by 2011.^2–6^ Responding to this epidemic,^6^ Florida implemented a 2011 law to reduce opioid prescribing at rogue pain clinics or “Pill Mills.”^7,8^ Although this law exempted patients with cancer and sickle cell disease, those patients experienced difficulty and stigma when filling prescriptions for opioid analgesics.^8,9^

By 2016, the Centers for Disease Control and Prevention (CDC) issued its “Guideline for Prescribing Opioids for Chronic Pain… to improve communication between clinicians and patients about the risks and benefits of opioid therapy for chronic pain, improve the safety and effectiveness of pain treatment, and reduce the risks associated with long-term opioid therapy, including opioid use disorder, overdose, and death [for] patients 18 years and older with chronic pain outside of active cancer treatment, palliative care, and end-of-life care.”^10,11^

Similarly, to further reduce improper opioid prescribing, Florida mandated queries of the state's prescription drug monitoring program (PDMP) for prescribing any controlled medication beginning July 1, 2018.^12–15^ This intensified concerns about stigma for patients with pain from cancer and sickle cell disease and prompted our first three-month quality improvement (QI) project to assure compliance with the new law.^13,14^ On September 6, 2018, Florida's PDMP reports first began including patients' overall overdose risk scores.^14–16^

Since then, policies intended to reduce opioid prescribing have resulted in inadvertent patient harms, including impaired medication access for patients with cancer pain^17–20^ and adverse events from excessively rapid or nonconsensual opioid tapers.^21–24^ In addition, critics of opioid therapy have cited a lack of long-term evidence for its effectiveness and safety.^25–27^ Providing detail-oriented, complex, high-level, and goal-concordant nonhospice palliative care to “persons living with serious, complex, and life-threatening illness”^28^ provided us with a different perspective of long-term opioid therapy.

In this QI project, we examined the effect of providing nonhospice palliative care over a four-year timeframe to outpatients with serious, complex illness who had cancer- or noncancer-related chronic pain or both. We offer the first evidence that outpatients with severe chronic pain who received opioid therapy within the context of nonhospice palliative care over the course of four years: (1) demonstrated durable lasting treatment-related reduction in pain intensity, (2) experienced preservation of their performance status during that timeframe, and (3) experienced reduction in their overall risk for fatal drug overdose over time.

## Measures

### Key metrics

All of the patients in this QI project were referred by oncologists and hematologists to the Palliative Medicine Clinic to manage chronic pain arising directly from active cancer, indirectly from cancer through pain syndromes from prior cancer treatment^29^ or from noncancer pain associated with hematologic conditions, primarily complications of sickle cell disease. Treatment-related reduction in pain intensity over time was one of the project's key metrics. At each of the patients' clinical encounters over the duration of the project, we recorded their self-assessed pain ratings using a 0-to-10 numerical rating scale.^30^ We defined treatment-related reduction in pain intensity as the difference between patients' self-assessed pain intensity with and without pain medications.

In addition, we assessed functional status for a more comprehensive clinical assessment.^31^ We recorded the patients' Karnofsky Performance Scores^32^ or similarly scaled Palliative Performance Scores (PPSs)^33^ during each clinical encounter over the project's duration. We employed 5-point increments when attending clinicians reported a range between two performance levels; for example, a PPS assessed as 70 to 80 was recorded as a value of 75.

We also recorded patients' overall overdose risk scores “to identify potential risk for unintentional fatal opioid overdose” from patients' PDMP reports.^34–36^ Notably, these scores were calculated using a proprietary tool purchased and broadly used by state PDMPs for benchmarking this metric.^34–36^ Risk factors used to calculate the overall overdose risk score include, but are not limited to, the number prescribers for a patient, the “number of pharmacies at which a patient fills medications, the amount or strength of medication being prescribed, [and] the amount of additional medications (if any) that may increase the potency (or risk) of other medications.”^37^ Exactly how these and other, unknown factors combine to create the final proprietary overdose risk score remains unclear.^34–37^

When analyzing these key metrics, we graphed them to confirm a normal distribution visually. We employed a two-tailed paired *t* test to compare means for data gathered from the cohort group in 2018 and 2022, with *p* < 0.05 considered statistically significant.

### Secondary metrics

In addition to these key metrics, we also recorded the Florida PDMP's value for the patients' average daily opioid use in morphine milligram equivalents (MME), because a 90 MME dosing threshold “has been used to justify abruptly stopping opioid prescriptions or coverage,”^17^ although with associated unanticipated patient harms.^17–20,22–24,38–42^ Unfortunately, average MME was not reported a consistently as a metric in Florida PDMP queries during 2018, which prevented a comparison over time.

We also categorized cohort members by type of chronic pain—whether from active cancer, pain in cancer survivors,^29^ or noncancer pain—noting that not only has opioid prescribing guidance varied significantly for these pain types for more than two decades,^10,11,43–50^ but their very definitions have varied, as well.^29,31^ Finally, we recorded patients' demographic information to more fully characterize the project cohort's composition in 2022.

## Intervention

### QI project cohort

**FIG. 1.**
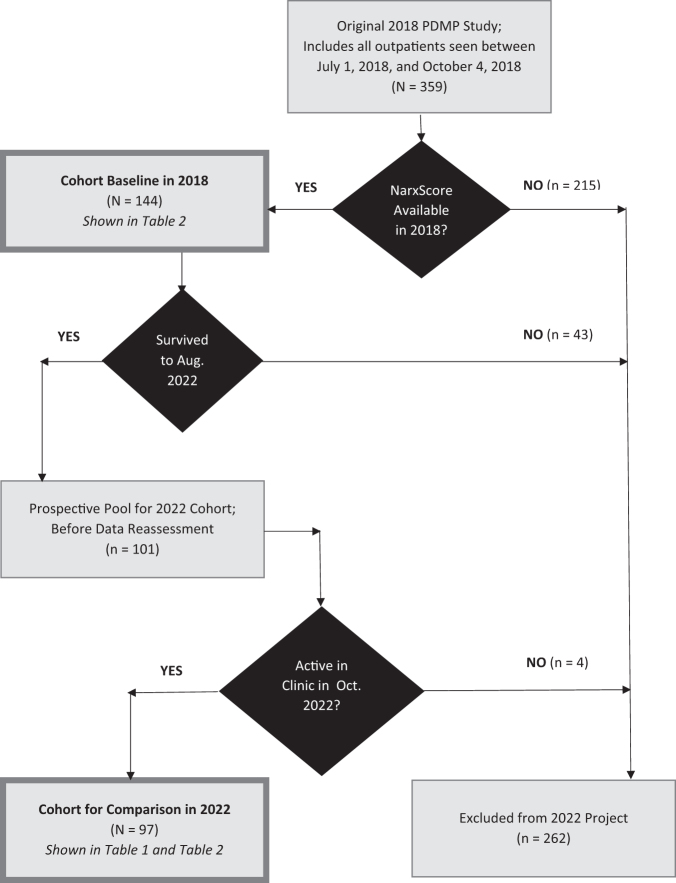
Identification of the project cohort in 2018 and 2022.

The project cohort is a subgroup from a 2018 QI database of all mandatory PDMP queries performed in the outpatient palliative medicine clinic between July 1, 2018, and October 4, 2018,^13,14^ as shown in Figure 1. To study this cohort over time, we included patients on whom we were able to record overall overdose risk scores in 2018, who were still receiving palliative care through October 2022. Before reassessing queries for this 2022 QI cohort, the Orlando Health Institutional Review Board (IRB) determined that any results should be published as a QI project, not as human subject research.^51^ We performed PDMP queries for all qualifying patients prospectively between August 1, 2022 and October 31, 2022.

### Clinical setting and guidelines for pain care

Over four years, the cohort received palliative care longitudinally at a hospital-based outpatient palliative medicine clinic embedded within a regional cancer center on the central campus of Orlando Regional Medical Center (ORMC), an 808-tertiary hospital in downtown, Orlando, Florida.^52^ All patients were referred by oncologists or hematologists to manage chronic pain, with most coming from Orange, Seminole, Lake, and Osceola counties, a population of >2.5 million people.^53^

Clinical encounters were conducted face-to-face between an attending clinician and the patient and/or any family members in the outpatient palliative medicine clinic. We established individualized goals for pain care through informed discussions that emphasized patient autonomy and balanced benefits and risks of opioid therapy. We sought goal-concordant reduction in pain intensity sufficient to allow for patients' participation in their desired activities with minimal side effects from pain care, including opioid therapy. Time between clinical encounters varied somewhat, but patients visited the clinic at least every three months, if not more frequently.

The attending clinician was a physician with board certification in internal medicine and HPM with >20 years of clinical experience in outpatient palliative care, or a certified nurse practitioner with five years of experience in outpatient palliative care and a decade of inpatient oncology experience. The weekly clinic template allowed for 10 new and 42 follow-up visits for the physician or 60 follow-up visits for the advance practice nurse. Patients received supplementary outpatient services from other multidisciplinary team members at other outpatient offices in ORMC's Downtown Campus based on their individual needs, including social work, pastoral care, physical and occupational therapy, mental health counseling, wound management, medical subspecialty, and community support services.

Pain care was provided to the patients in accordance with Florida laws^12,15,54^ and guidelines for treating pain in active cancer,^45^ cancer survivors,^10,11,46,47^ sickle cell disease,^48^ or for providing palliative care.^49^ Notably, Florida does not require urine drug testing in the population of patients seen in our clinic,^12^ which is consistent with Center to Advance Palliative Care (CAPC) educational recommendations for providing pain management in a palliative care setting.^55^

We assessed for aberrant opioid use or behaviors^56^ at each clinical encounter using a method described by a former clinical affiliate.^57^ We used “a combination of patient information, behavior patterns, and useful assessment tools,” including an assessment of adherence to the patients' pain regimen, clarifying any deviations from their prescribed dose.^57,58^ This included screening for positive responses to the Revised Opioid Tool during patient interviews and reviews of the electronic medical record.^59,60^ We also utilized the results of PDMP queries to identify other prescribers, which prompted discussions clarifying those prescriptions.^58^ We did not routinely perform urine drug screens, but implemented behavioral contracts, including urine drug testing, for patients with persistent aberrancy.^56,58^

## Outcomes

We identified 144 potentially qualifying patients from the 2018 QI study, but 43 (30%) died from their primary illnesses before the reassessment in this QI project; their cause of death was established by a review of death notes from their electronic medical record. Three patients moved out of the greater Orlando area and were lost to follow-up. No patients died from drug overdoses, but two patients exhibited aberrant opioid use^56^; one was consensually dismissed from the clinic after nonadherence to a behavioral agreement for repeated recreational cocaine use, whereas the other adhered to their agreement and consensual adjustments to the lowest effective opioid dose for controlling cancer pain.

Table 1 shows the demographic characteristics of the study cohort, which represented a total of 97 patients as of October 31, 2022. The cohort had a mean age of 58 years with 58% of the patients identifying as female. Only 29% of the cohort had pain from active cancer in 2022, whereas 57% were cancer survivors with pain syndromes from their cancer treatment. The most common pain syndrome in cancer survivors were radiation- or chemotherapy-induced neuropathies (29%), joint pain from aromatase inhibitor therapy or chemotherapy-related arthropathy (18%) and chronic pelvic or rectal pain after radiation therapy (16%).

**Table 1.** Cohort Characteristics in 2022 (*n* = 97)

Characteristic	Cohort result
Mean, median age (in years)	58, 61
Self-reported gender identity	
Male, *n* (%)	41 (42%)
Female, *n* (%)	56 (58%)
Type of pain	
Pain from active cancer, *n* (%)	28 (29%)
Pain in cancer survivor, *n* (%)	55 (57%)
Other chronic nonmalignant pain, *n* (%)	14 (14%)
Patients reporting reduced pain with opioid therapy, *n* (%)	94 (97%)
MME	
Mean, median MME/day (in mg/day)	351, 117
Patient receiving >90 MME/day, *n* (%)	53 (55%)
Range of MME/day (in mg/day)	0–2355

MME, morphine milligram equivalents.

The remaining 14% of the patients had chronic nonmalignant pain from hematologic conditions, and 83% of those patients had chronic severe bone pain from sickle cell disease. There was no statistically significant difference in mean reduction in pain intensity based on the patients' type of pain. The mean dose of opioid analgesia within the cohort was 351 MME/day with a median dose of 117 MME/day; notably, 53 patients (55%) had doses exceeding 90 MME/day. Ninety-four of 97 patients (97%) reported reduction in pain with opioid therapy.

Table 2 shows that the cohort exhibited a statistically significant durable treatment-related reduction in pain intensity from opioid therapy for a duration of four years. There was an average reduction in pain intensity of 4.9 points out of 10 with treatment, which was statistically unchanged from 2018. Treatment-related reduction in pain intensity ranged from 0 to 7.5 points out of 10. There was also a 2-point improvement in function scores over time (*p* = 0.01) that, although statistically significant (*p* = 0.01), may not have been meaningful clinically because the performance scales use a 10-point increment to define difference in function.

**Table 2.** Assessment of Quality Improvement Metrics Over Time

QI metric (scale range)	Cohort baseline 2018	Cohort reassessment 2022	*p*
Mean reduction in pain rating (0–10)	4.8	4.9	0.51
Mean performance status (0–100)	72	74	0.01
Mean overdose risk score (0–999)	383	312	<0.001

QI, quality improvement.

We interpreted our result to suggest stability in performance status in a cohort of chronically ill patients who would be reasonably expected to decline over time. Finally, Table 2 also shows a statistically significant reduction in overdose risk score of about 70 points over time (*p* < 0.001), offering the first evidence that providing opioid therapy as part of ongoing outpatient palliative care over time may mitigate opioid overdose risk. For context, given the proprietary nature of the overdose risk score, the risk of unintentional overdose death approximately doubles for every 100-point increase in the overdose risk score.^16^

## Limitations

We limited our QI study cohort to patients who were receiving palliative care for chronic pain in an outpatient clinic for a regional cancer center at a tertiary care hospital, so our results may not apply to a general population of patients with chronic pain. Definitively characterizing the cohort members' different types of chronic pain proved elusive due to a “lack of consensus regarding the definition of ‘cancer survivor’”^31^ and a lack of certainty about classifying the pain of patients with imaging evidence of treated metastatic disease, but no clinical or imaging evidence of “active” cancer. It is also unclear whether providing palliative care at a generalist level would produce similar results for patients receiving specialty level nonhospice palliative care.

We used overall risk scores to estimate overdose risk, following several states' reasoning for including that metric in their PDMPs.^13,16,34–36^ We did not examine other factors that might have influenced or reduce overdose risk over time, so we cannot conclusively infer that the provision of palliative care caused the reduction in risk observed in our project.

Also, because there was not a methodologically consistent determination of average daily MME by the Florida PDMP in 2018, we were unable to offer a dosing comparison for the cohorts' opioid use over time. We did not examine quality of life measures outside of pain relief and performance status, although these would be valuable metrics for future studies. Similarly, we did not examine long-term side effects of opioid therapy, such as endocrinopathies,^61^ which would have required diagnostic laboratory assessments and patient costs outside the scope of this QI project.

## Conclusions/Lessons Learned

This QI project presents evidence that providing ongoing palliative care over a four-year timeframe offers through lasting treatment-related reduction in pain intensity, preservation of performance status, and reduction in overall overdose risk. In the past, ethical challenges and restrictions on study durations limited evidence to support opioid therapy for treating chronic pain in the long term.^62–64^ Furthermore, we believe this project represents the longest-term prospective cohort assessment of opioid therapy for chronic pain examining these key metrics.^65–72^ In addition to demonstrating improved care, this QI project has important implications for national opioid policy.

Policies intended to decrease overdose deaths through reduced opioid prescribing have increased patient harms,^17–20,23,24,38–42^ including reduced access to analgesics for patients with cancer.^18–20^ Although reduced opioid prescribing has not reduced opioid-involved overdose deaths,^6^ naloxone co-prescribing offers an effective option for opioid overdose risk reduction.^73,74^ We have embraced a non-hospice palliative care (NHPC)-oriented^28^ “appropriate prescribing” approach to risk reduction that uses “regular assessment, treatment planning, and monitoring to provide effective pain control while avoiding addiction, abuse, overdose, diversion, and misuse.”^75^ We encourage others to similarly incorporate clinical principles for managing opioid therapy in ongoing palliative care to replicate and expand on the evidence from this QI project.^45–49,76^

Our findings also call attention to the common policy practice of distinguishing cancer from noncancer pain and begs the question of “whether it is science or politics that that demands there be a difference.”^77^ Although both medical literature and health policy commonly distinguish cancer and noncancer pain,^10–12,18,20,29,31,43,50^ “a patient with pain from a cancer etiology has no different physiology than a patient with pain of noncancer etiologies.”^78^ Furthermore, whereas the 2016 CDC Guideline applied to “cancer survivors with chronic pain who have completed cancer treatment, are in clinical remission, and are under cancer surveillance only,”^10,11^ the revised 2022 CDC Guideline has acknowledged pain in survivors as cancer-related pain.^50^ Our project findings support criticism of treating chronic cancer- and noncancer-related pain differently,^77^ and we hope they will prompt corrective introspection by educators and policymakers alike.

Finally, by 2020, 39 states had imposed enforceable limits on opioid prescribing, including 5 states that restricted daily doses to 90 MME or less.^79^ More than half of the patients in our cohort would have been ineligible to receive their effective opioid dose in those five states, affirming that “some patients with acute or chronic pain can benefit from taking opioid pain medications at doses greater than generally recommended in the [2016 CDC Guideline] and that such care may be medically necessary and appropriate.”^80^

Each state controls its own laws and policies that limit opioid prescribing,^81^ and these policies should ideally “protect the public and improve the quality, safety, and integrity of health care.”^82^ Our project showed that ongoing palliative care can maintain health care quality and reduce risks of opioid therapy, which supports reassessing some restrictive opioid prescribing laws. We remain hopeful that this QI project will encourage similar research that will lead to regulatory and legal changes needed to “strike the proper balance [in]… maintaining legitimate patient access to needed medications”^83^ while reducing risks for patient harms.
